# Metallic nickel nano- and fine particles induce JB6 cell apoptosis through a caspase-8/AIF mediated cytochrome *c*-independent pathway

**DOI:** 10.1186/1477-3155-7-2

**Published:** 2009-04-20

**Authors:** Jinshun Zhao, Linda Bowman, Xingdong Zhang, Xianglin Shi, Binghua Jiang, Vincent Castranova, Min Ding

**Affiliations:** 1 Pathology and Physiology Research Branch, Health Effects Laboratory Division, National Institute for Occupational Safety and Health, Morgantown, WV, 26505, USA; 2 Graduate Center for Toxicology, College of Medicine, the University of Kentucky, Lexington, KY, 40515, USA; 3 Department of Microbiology, Immunology, and Cell Biology, West Virginia University, Morgantown, WV, 26505, USA

## Abstract

**Background:**

Carcinogenicity of nickel compounds has been well documented. However, the carcinogenic effect of metallic nickel is still unclear. The present study investigates metallic nickel nano- and fine particle-induced apoptosis and the signal pathways involved in this process in JB6 cells. The data obtained from this study will be of benefit for elucidating the pathological and carcinogenic potential of metallic nickel particles.

**Results:**

Using 3-(4,5-dimethylthiazol-2-yl)-2,5-diphenyltetrazolium bromide (MTT) assay, we found that metallic nickel nanoparticles exhibited higher cytotoxicity than fine particles. Both metallic nickel nano- and fine particles induced JB6 cell apoptosis. Metallic nickel nanoparticles produced higher apoptotic induction than fine particles. Western-blot analysis showed an activation of proapoptotic factors including Fas (CD95), Fas-associated protein with death domain (FADD), caspase-8, death receptor 3 (DR3) and BID in apoptotic cells induced by metallic nickel particles. Immunoprecipitation (IP) western blot analysis demonstrated the formation of the Fas-related death-inducing signaling complex (DISC) in the apoptotic process. Furthermore, lamin A and beta-actin were cleaved. Moreover, we found that apoptosis-inducing factor (AIF) was up-regulated and released from mitochondria to cytoplasm. Interestingly, although an up-regulation of cytochrome *c *was detected in the mitochondria of metallic nickel particle-treated cells, no cytochrome *c *release from mitochondria to cytoplasm was found. In addition, activation of antiapoptotic factors including phospho-Akt (protein kinase B) and Bcl-2 was detected. Further studies demonstrated that metallic nickel particles caused no significant changes in the mitochondrial membrane permeability after 24 h treatment.

**Conclusion:**

In this study, metallic nickel nanoparticles caused higher cytotoxicity and apoptotic induction than fine particles in JB6 cells. Apoptotic cell death induced by metallic nickel particles in JB6 cells is through a caspase-8/AIF mediated cytochrome *c*-independent pathway. Lamin A and beta-actin are involved in the process of apoptosis. Activation of Akt and Bcl-2 may play an important role in preventing cytochrome *c *release from mitochondria to the cytoplasm and may also be important in the carcinogenicity of metallic nickel particles. In addition, the results may be useful as an important reference when comparing the toxicities of different nickel compounds.

## Background

Nickel is a widely distributed metal that is industrially applied in many forms. The high consumption of various nickel products inevitably leads to occupational and environmental pollution [1]. Carcinogenicity of nickel compounds has been well documented [2-4]. However, the carcinogenic effect of metallic nickel is still unclear [5]. Evidence indicates that various nickel compounds, but not metallic nickel, cause pulmonary inflammation, fibrosis, emphysema, and cancer [6]. The International Agency for Research on Cancer (IARC), therefore, classified all nickel compounds as human carcinogens in 1990 [7]. The available epidemiological studies on the carcinogenicity of metallic nickel are limited by inadequate exposure information, low exposures, short follow-up periods, and small numbers of cases [8]. But evidences from studies in experimental animals suggest that metallic nickel is reasonably anticipated to be a human carcinogen [5].

The metallic nickel nanoparticle is a product with many new characteristics, which include a high level of surface energy, high magnetism, low melting point, high surface area, and low burning point. Therefore, it can be widely used in modern industries [9]. However, these same properties of metallic nickel nanoparticles may present unique potential health impact [10]. Based on the fact that TiO_2 _nanoparticles are more toxic than TiO_2 _fine particles [11], the pathological effects of nickel compounds and metallic nickel may also depend on their particle size. Nickel compound (acetate)-induced apoptosis has been reported in Chinese hamster ovary cells [12] and T cell hybridoma cells [13]. But the mechanism of cell death induced by metallic nickel nano- and fine particles has not been clearly elucidated.

Apoptosis is a highly regulated process that is involved in pathological conditions [14]. Diseases may be caused by a malfunction of apoptosis. An inefficient elimination of mutated cells may favor carcinogenesis [15]. However, excessive apoptosis was shown to contribute to pulmonary fibrosis in mice [16]. Furthermore, enhanced apoptosis may indirectly trigger compensatory cell proliferation to ensure tissue homeostasis and promote the fixation of mutagenic events. Evidence indicates that apoptosis is also involved in pulmonary disorders, such as acute lung injury, diffuse alveolar damage, and idiopathic pulmonary fibrosis [16,17]. Therefore, the apoptotic properties may be important in the mechanisms of pathogenicity and carcinogenicity induced by the metallic nickel particles.

Accordingly, the aim of the present study is to compare the cytotoxicity and apoptosis induced by metallic nickel nano- and fine particles, and to elucidate the mechanisms of cell death induced by these particles *in vitro*.

## Methods

### Materials

Metallic nickel nanoparticles, average size 80 nm, were purchased from Inframat Advanced Materials, LLC (Farmington, CT). Metallic nickel fine particles, average size of 3 μm, were purchased from Sigma-Aldrich (Milwaukee, WI). Eagle's minimal essential medium (EMEM) was obtained from Lonza (Walkersville, MD). Fetal bovine serum (FBS), trypsin, pencillin/streptomycin and L-glutamine were purchased from Life Technologies, Inc. (Gaithersburg, MD). YO-PRO-1 [YP, 1 mM solution in dimethyl sulfoxide (DMSO)] and propidium iodide (PI, 1.0 mg/ml in water) were purchased from Invitrogen (Carlsbad, CA). Anti-h/m caspase-8 antibody was obtained from R&D systems (Minneapolis, MN). Total Akt (Akt), phospho-Akt (p-Akt, ser 473), BID, and cleaved caspase-3 antibodies were purchased from Cell Signaling Technology (Beverley, MA). All other antibodies were obtained from Santa Cruz Biotechnology Co. (Santa Cruz, CA). Cell proliferation kit I (MTT assay kit) was obtained from Roche Applied Science (Penzberg, Germany). Mitochondria Staining Kit was purchased from Sigma-Aldrich (Saint Louis, MO).

### Preparation of metallic nickel nano- and fine particles

Stock solutions of metallic nickel nano- or fine particles were prepared by sonification on ice using a Branson Sonifier 450 (Branson Ultrasonics Corp., Danbury, CT) in sterile PBS (10 mg/ml) for 30 sec, then kept on ice for 15 sec and sonicated again for a total of 3 min at a power of 400 W. Before use, these particles were diluted to a designed concentration in fresh culture medium. All samples were prepared under sterile conditions.

### Surface area and size distribution measurements

Surface area of metallic nickel particles was measured using the Gemini 2360 Surface Area Analyzer (Mircomeritics; Norcross, GA) with a flowing gas technique according to the manufacturer's instructions. The size distribution of metallic nickel particles was detected using scanning electron microscopy (SEM). Briefly, metallic nickel particles were prepared by sonification. Then, the samples were diluted in double-distilled water and air dried onto a carbon planchet. Images were collected on a scanning electron microscope (Hitachi S-4800; Japan) according to the manufacturer's instructions. Optimas 6.5 image analysis software (Media Cybernetics; Bethesda, MD) was used to measure the diameter of metallic nickel particles.

### Cell culture

Mouse epidermal JB6 cells were maintained in 5% FBS EMEM containing 2 mM L-glutamine and 1% penicillin-streptomycin (10,000 U/ml penicillin and 10 mg/ml streptomycin) at standard culture conditions (37°C, 80% humidified air, and 5% CO_2_). For all treatments, cells were grown to 80% confluence.

### Cytotoxicity assay

Cytotoxicity of metallic nickel nano- or fine particles to JB6 cells was assessed by a MTT assay kit following the manufacturer's instructions. Briefly, cells were plated in 100 μl EMEM at a density of 10^4 ^cells/well in a 96 well plate. The cells were grown for 24 h and treated with various concentrations of metallic nickel particles. After 24 h incubation, 10 μl MTT labeling reagent was added in each well and the plates were further incubated for 4 h. Afterward, 100 μl solubilization solution was added to each well and the plate was incubated overnight at 37°C. The optical density (OD) of the wells was measured at a wavelength of 575 nm with reference of 690 nm using an ELISA plate reader. Results were calibrated with OD measured without cells.

### Detection of apoptosis

YP staining was used to determine if cell death induced by metallic nickel particles was apoptotic. Briefly, JB6 cells were seeded onto a 24-well plate overnight. Then, cells were treated with/without various concentrations of metallic nickel nano- or fine particles for 24 h. Before microscopy, YP was added into the cultures (10 μg/ml) for 1 h. Then, cells were washed two times with EMEM medium. Apoptotic cells were monitored using a fluorescence microscope (Axiovert 100 M; Zeiss, Germany). Percentage of cells exhibiting apoptosis was calculated.

### Identification of apoptosis

Dual staining using YP and PI was used to distinguish between apoptosis and necrosis as described by Debby and Boffa [18,19] with some modifications. JB6 cells were seeded onto a 24-well plate and incubated overnight. Then, cells were treated with/without various concentrations of metallic nickel nano- or fine particles. One hour later, YP and PI were added into the cultures with a final concentration of 10 μg/ml and 1 μM, respectively. The progression of cell death in the living cultures was monitored at different time points on a fluorescence microscope (Axiovert 100 M). YP stained cells were detected with blue excitation filter. PI stained cells were measured by green excitation filter.

### Western blot analysis

Briefly, cells were plated onto a 6-well plate. The cultures were grown 24 h and then starved in 0.1% FBS EMEM overnight. Cells were treated with/without metallic nickel nano- or fine particles. After treatment, the cells were extracted with 1× SDS sample buffer supplemented with protease inhibitor cocktail (Sigma-Aldrich). Protein concentrations were determined using the bicinchoninic acid method (Pierce; Rockford, IL). Equal amounts of proteins were separated by 4–12% Tris glycine gels. Immunoblots for expression of Fas, FADD, caspase-8, DR3, death receptor 6 (DR6), tumor necrosis factor-receptor 2 (TNF-R2), caspase-3, caspase-6, caspase-9, BID, cleaved BID, Bcl-2, BAX, cytochrome *c*, AIF, beta-actin, and lamin A were detected. Experiments were performed three or more times, and equal loading of protein was ensured by measuring total Akt, and alpha- or beta-tubulin expression.

To prepare the subcellular fractionation, cells were washed twice with cold PBS. Then, cells were lysed in 100 μl of cold isolation buffer A (20 mM Hepes/10 mM KCl/1.5 mM MgCl_2_/1 mM EDTA/1 mM EGTA/1 mM DTT) supplemented with protease inhibitor cocktail and 250 mM sucrose. After incubating on ice for 15 min, the cells were broken by passing through 22-gauge needles 25 times. The lysate was centrifuged at 800 × g for 5 min to remove unbroken cells and nuclei. The supernatant was then re-centrifuged (10,000 × g, 30 min, 4°C) to obtain a pellet. The resultant supernatant was the cytosolic fraction and the pellet contained mitochondria. The cytosolic fraction was diluted using 100 μl of 2× SDS sample buffer. The mitochondrial pellet was resuspended in 1× SDS sample buffer.

### IP western blot analysis

After treatment, JB6 cells were lysed in buffer B (20 mM Tris-HCl, pH 7.5, containing 150 mM NaCl, 2 mM EDTA, 1% Triton X-100, 10% glycerol, and 10 μl/ml protease inhibitor cocktail) for 15 min at 4°C. Lysates were centrifuged at 25,000 × *g *for 15 min. Protein concentrations of the supernatants were determined. Equal amounts of proteins were immunoprecipitated overnight with rabbit anti-caspase-8 antibody (1:200) at 4°C. The supernatant was further incubated with 20 μl of protein A/G-agarose slurry for 3 h at 4°C. Beads were pelleted, washed three times in buffer B, and finally boiled in 1× SDS sample buffer. Proteins were separated by 4–12% Tris glycine gels. Fas and FADD proteins were detected as described in western blot analysis.

### Detection of mitochondrial membrane permeability

JB6 cells were seeded onto a 24-well plate overnight. Cells were treated with/without metallic nickel nano- or fine particles for 24 h. Changes of mitochondrial membrane permeability were evaluated using a mitochondrial staining kit (JC1 staining) according to the manufacturer's instructions. Briefly, a staining mixture was prepared by mixing the staining solution with an equal volume of the EMEM medium. Cells were incubated in the staining mixture (0.4 ml/well) for 30 min at 37°C in a humidified atmosphere containing 5% CO_2_. Thereafter, cells were washed two times in medium, followed by addition of fresh medium. Mitochondrial membrane permeability was monitored on a fluorescence microscope (Axiovert 100 M).

### Statistical analysis

Data are presented as means ± standard errors (S.E.) of n experiments/samples. Significant differences were determined using R software or the Student's *t*-test. Significance was set at *p *≤ 0.05.

## Results

### Surface area and size distribution of metallic nickel particles

To measure the surface area and size distribution of nickel particles, Gemini 2360 Surface Area Analyzer and scanning electron microscopy were used, respectively. The average surface area of metallic nickel nanoparticles was 4.36 m^2^/g compared to 0.40 m^2^/g for fine particles. The average size distribution of metallic nickel nano- and fine particles is 92.32 nm and 3.34 μm, respectively (Table 1).

**Table 1:** Surface area and size distribution of metallic nickel particles

	Nickel fine particles	Nickel nanoparticles
		
Surface area (m^2^/g)	0.4 ± 0.01	4.36 ± 0.02
Average size	3.34 ± 0.67 (μm)	92.32 ± 29.69 (nm)

Surface area was determined by gas absorption and particle size by scanning electron microscopy. Values are means ± S.E. of six independent assays.

### SEM images of the metallic nickel particles

**Figure 1:**
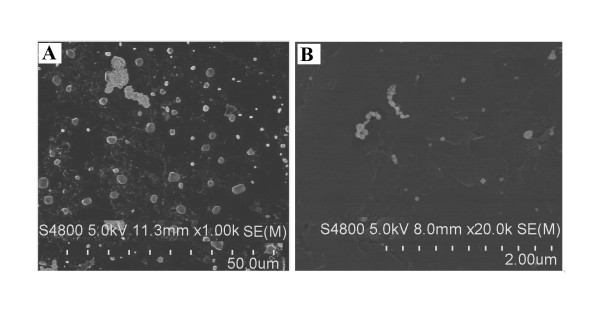
**SEM images of metallic nickel particles**. SEM images of metallic nickel fine (**A**) or nanoparticles (**B**) were captured on a scanning electron microscope.

Metallic nickel nano- or fine particles were prepared by sonification. Then, the samples were diluted in double-distilled water and air dried onto a carbon planchet. SEM images were captured on a scanning electron microscope (Figure 1A and 1B).

### Effects of metallic nickel particles on cell viability and apoptotic induction

**Figure 2:**
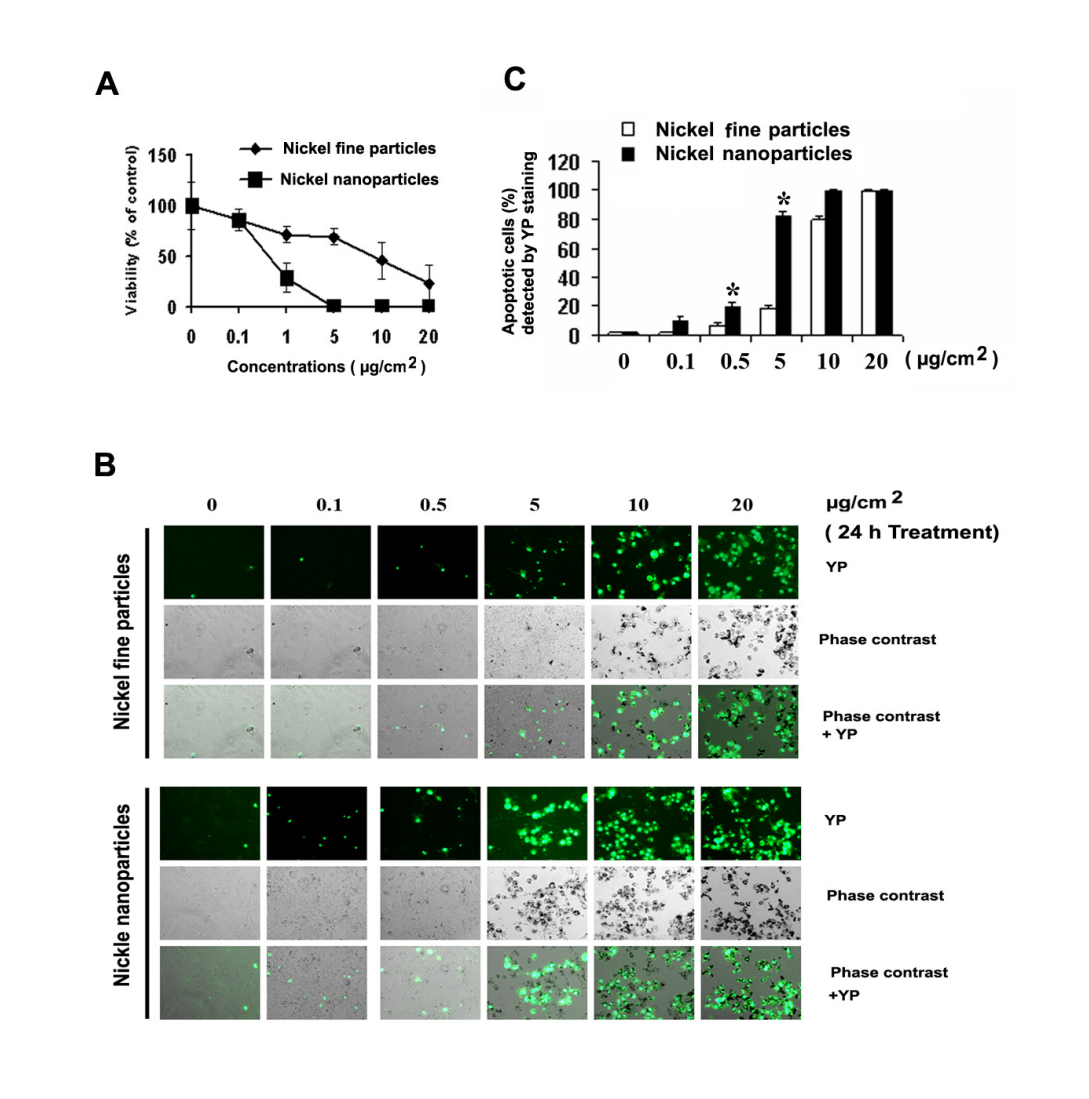
**Effects of metallic nickel particles on cell viability and apoptotic induction**. JB6 cells were exposed to various concentrations of metallic nickel nano- or fine particles for 24 h. Cell viability was detected by MTT assay. Significantly less viability was observed in cells treated with nanoparticles compared to fine particles analyzed by R software (*p *< 0.05). Data shown are means ± S.E. of four independent assays (**A**). Apoptosis induced by metallic nickel nano- or fine particles was detected by YP staining (**B**, 10× magnification). Metallic nickel nanoparticles induced more apoptosis than fine particles at 0.5 and 5 μg/cm^2 ^analyzed by Student's *t*-test (*p *< 0.05) indicated by * (**C**). Data shown are means ± S.E. of three independent assays.

To determine whether there is a difference in the cytotoxicity induced by different sizes of metallic nickel particles, various concentrations (0.1–20 μg/cm^2^) of metallic nickel nano- or fine particles were used to study the effects on cell viability in JB6 cells by MTT assay. Treatment of JB6 cells with metallic nickel particles for 24 h caused a dose-dependent cytotoxicity (Figure 2A). Cytotoxicity induced by metallic nickel nanoparticles was significantly higher than that induced by fine particles.

To study the apoptosis induced by metallic nickel nano- or fine particles, YP staining was used. JB6 cells were treated with various concentrations of metallic nickel nano- or fine particles from 0.1 to 20 μg/cm^2 ^for 24 h. Results indicated that both metallic nickel nano- and fine particles induced JB6 cell apoptosis (Figure 2B). The percentages of apoptotic cells were significantly higher in cells treated with nanoparticles than fine particles between the concentration of 0.5 and 5 μg/cm^2 ^(Figure 2C). At the concentration 5 μg/cm^2^, there was a 4-fold increase in apoptosis induced by nanoparticles compared to fine particles.

### Identification of apoptosis induced by metallic nickel particles

**Figure 3:**
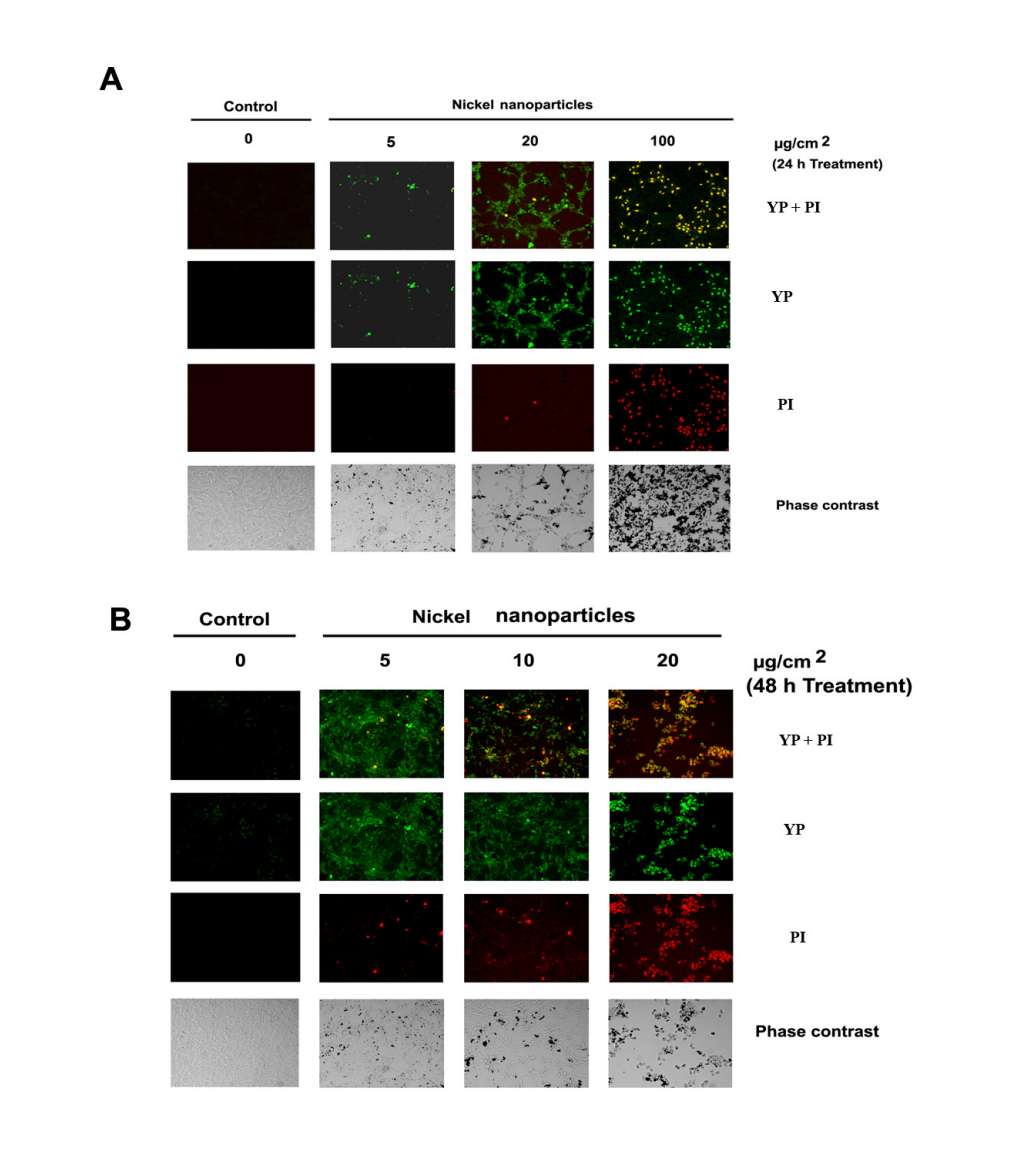
**Identification of apoptosis induced by metallic nickel nanoparticles**. JB6 cells were seeded onto 24-well plate and incubated overnight. Cells were treated with/without metallic nickel nanoparticles. Continuous monitoring of apoptosis and necrosis was conducted by using a dual fluorescence dye assay after 24 h treatment (**A**) or 48 h treatment (**B**).

To distinguish between apoptosis and necrosis induced by metallic nickel nano- or fine particles, a dual staining assay using YP and PI was applied. The results showed that both metallic nickel nano- and fine particles (data not shown) could induce JB6 cell apoptosis demonstrated by the positive staining of YP at an early exposure time (24 h) in a dose range of 0.1–20 μg/cm^2 ^(Figure 3A). Enhanced dose (100 μg/cm^2^, 24 h) or extended treatment (20 μg/cm^2^, 48 h) resulted in necrosis or late apoptosis demonstrated by the positive staining of both YP and PI (Figure 3A and 3B).

### Effects of metallic nickel particles on caspase-8, Fas, FADD, DR3, DR6, TNF-R2, p-Akt, DISC, lamin A, beta-actin, BID, Bcl-2, and BAX

**Figure 4:**
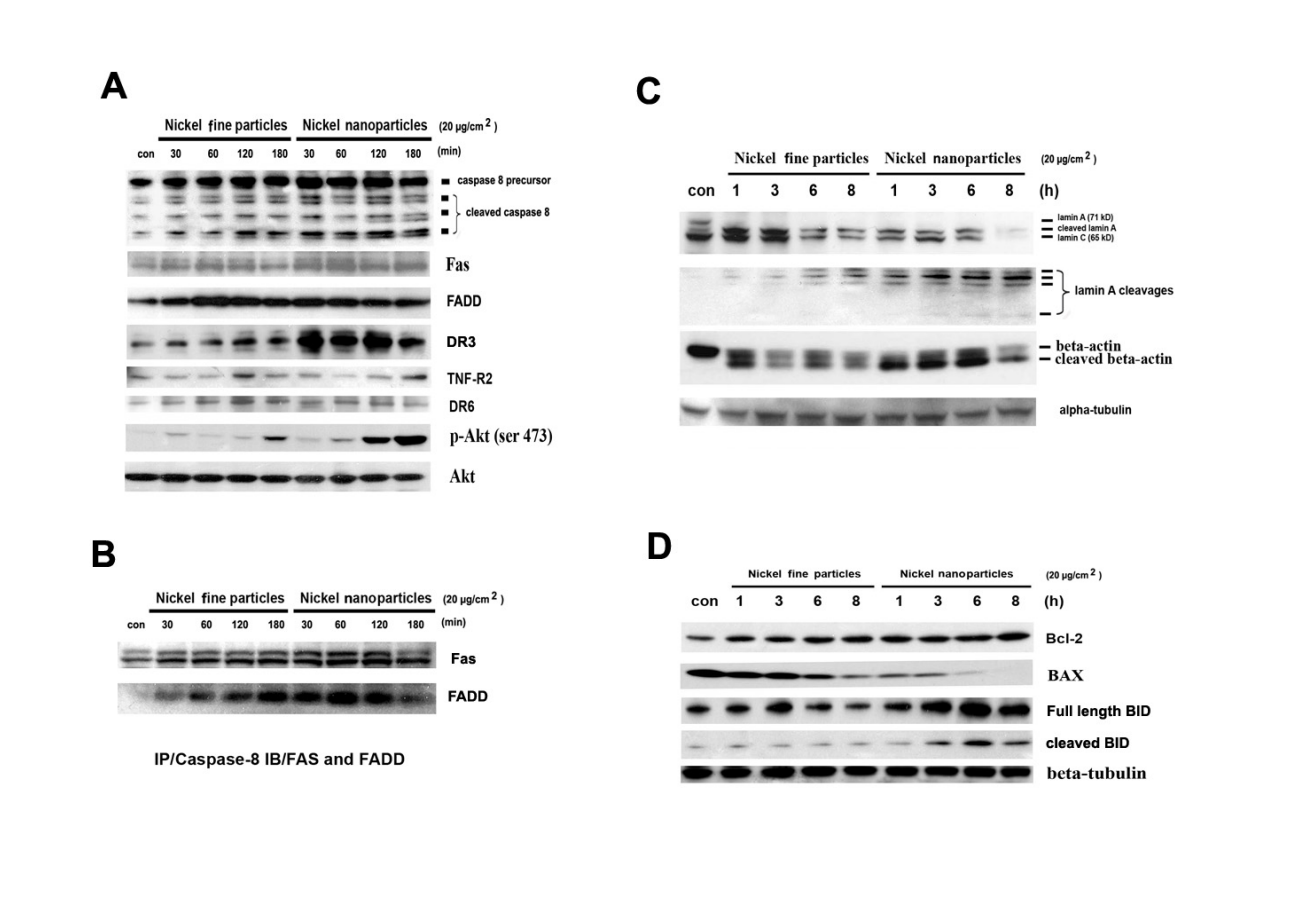
**Effects of metallic nickel particles on caspase-8, Fas, FADD, DR3, DR6, TNF-R2, p-Akt, DISC, lamin A, beta-actin, BID, Bcl-2, and BAX**. Cells were treated with 20 μg/cm^2 ^metallic nickel particles for 30, 60, 120, and 180 min. Expressions of caspase-8, Fas, FADD, DR3, DR6, TNF-R2, and p-Akt were analyzed by western blot (**A**). To investigate the formation of DISC, IP western blot was used (**B**). Cells were treated with metallic nickel particles for 1, 3, 6, and 8 h. Effects of metallic nickel particles on lamin A, beta-actin, and Bcl-2 family were detected by western blot (**C and D**).

Previous studies have demonstrated that apoptosis activates an upstream protease caspase-8 [20,21]. In this study, JB6 cells were treated with 20 μg/cm^2 ^of metallic nickel nano- or fine particles for 30, 60, 120, and 180 min. Protein expressions were detected by western-blot. Results indicated that caspase-8 was activated by these particles (Figure 4A).

Two important signals are known to be involved in apoptosis, which include the TNF and the Fas-Fas ligand-mediated pathways. Both involve the TNF receptor family coupled to extrinsic signals [22]. To investigate the involvement of extrinsic signals in the apoptotic process induced by metallic nickel particles, expression of the TNF family members of Fas, FADD, DR3, DR6, and TNF-R2 was examined. Results demonstrated that metallic nickel particles activated Fas, FADD and DR3. However, no obvious change was found in the protein expression of DR6 or TNF-R2 (Figure 4A).

Akt is a well-characterized member of PI3 kinase-mediated signaling pathways, regulating cell growth, apoptosis, as well as other cellular responses. Akt activation inhibits apoptosis by phosphorylating the Bcl-2 related proteins. In addition, Akt activation is sufficient to inhibit the release of cytochrome *c *from mitochondria and the alterations in the inner mitochondrial membrane potential [23]. In this study, results indicated that both metallic nickel nano- and fine particles induced Akt phosphorylation in a time-dependent manner (Figure 4A).

As caspase-8 activation was detected, we further determined the involvement of the DISC formation in the process of apoptosis induced by metallic nickel particles. The interaction between Fas and FasL results in the formation of the DISC, which consist of Fas, FADD, and caspase-8 [22]. To investigate the formation of DISC, IP western blot was used. JB6 cells were treated with 20 μg/cm^2 ^metallic nickel nano- or fine particles for 30, 60, 120, and 180 min. Anti-caspase-8 IP revealed an interaction of Fas and FADD with caspase-8, demonstrating DISC formation and the initiation of Fas-induced apoptotic pathway (Figure 4B).

The cellular morphology associated with the apoptotic process has been well characterized by membrane blebbing, formation of apoptotic bodies, and chromosome condensation. These apoptotic changes are the result of the cleavage of cellular proteins, such as lamin and actin [24,25]. In this study, JB6 cells were treated with 20 μg/cm^2 ^metallic nickel nano- or fine particles for 1, 3, 6, and 8 h. Western blot revealed that the cleavages of lamin A and beta-actin were detected as early as 1 h post-exposure. Both particles induced lamin A cleavages in a time-dependent manner (Figure 4C).

BID, a proapoptotic member of the Bcl-2 family, is a physiological substrate of caspase-8 which causes mitochondrial damage [26]. The results demonstrated that metallic nickel nano- or fine particles induced BID cleavage in a time-dependent manner. Interestingly, Bcl-2, an anti-apoptotic protein, was up-regulated. BAX, a proapoptotic member of Bcl-2 family, was down-regulated (Figure 4D).

### Effects of metallic nickel particles on AIF, cytochrome c, caspase-3, -6, and -9

**Figure 5:**
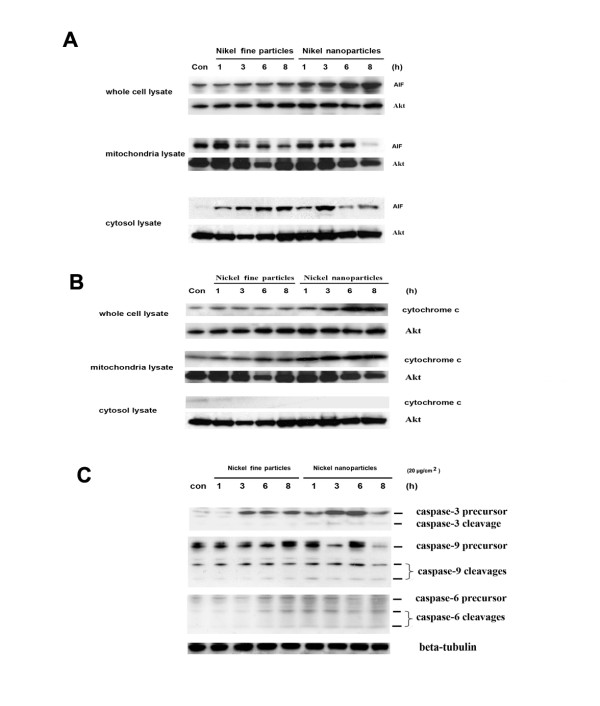
**Effects of metallic nickel particles on AIF, cytochrome *c*, and caspase-3, -6, and -9**. To determine the effects of metallic nickel particles on AIF, cytochrome *c*, and caspase-3, -6, and -9, JB6 cells were seeded onto a 6-well plate. After 24 h incubation, cells were starved in 0.1% FBS EMEM overnight. Then, cells were treated with 20 μg/cm^2 ^metallic nickel particles for 1, 3, 6, and 8 h. Western blot analysis was used to detect the effects of metallic nickel particles on AIF (**A**), cytochrome *c *(**B**), and caspase-3, -6, and -9 (**C**).

AIF is a recently characterized proapoptotic mitochondrial protein [27]. It is normally confined to the mitochondrial inter membrane space. After release from mitochondria into the cytoplasm, AIF can stimulate cell apoptosis [28]. To test the effects of metallic nickel particles, JB6 cells were treated with 20 μg/cm^2 ^nano- or fine particles for 1, 3, 6, and 8 h. Western blots revealed that both nano- and fine particles induced mitochondrial AIF up-regulation and release from mitochondria to the cytoplasm after 1 h treatment (Figure 5A).

Cytochrome *c *is an important apoptotic factor in the intrinsic apoptotic pathway which is released into the cytoplasm from the mitochondria in response to proapoptotic stimuli [29]. To investigate the possible involvement of cytochrome *c *release in the process of apoptosis induced by metallic nickel particles, JB6 cells were treated with 20 μg/cm^2 ^of metallic nickel nano- or fine particles for 1, 3, 6, 8 h. Western blot analysis indicated that cytochrome *c *was not released from the mitochondria into the cytoplasm although metallic nickel particles could induce cytochrome *c *up-regulation (Figure 5B).

Caspases are a family of cysteine proteases which play essential roles in apoptosis, necrosis and inflammation [30]. Eleven caspases have so far been identified in humans. There are two types of apoptotic caspases: initiator caspases and effector caspases. Initiator caspases (e.g. caspase-8) cleave inactive pro-forms of effector caspases, thereby activating them. Effector caspases (e.g. caspase-3 and -6) in turn cleave other protein substrates resulting in the apoptotic process. Since activation of caspase-8 was detected, we next examined the possible involvement of caspase-3, -6, and -9 in the process of apoptosis induced by metallic nickel particles. Results indicated that metallic nickel particles induced only a slight activation of caspase-3, -6, and -9. Interestingly, caspase-3 precursor was significantly up-regulated by metallic nickel particles (Figure 5C).

### Effects of metallic nickel particles on mitochondrial membrane permeability

**Figure 6:**
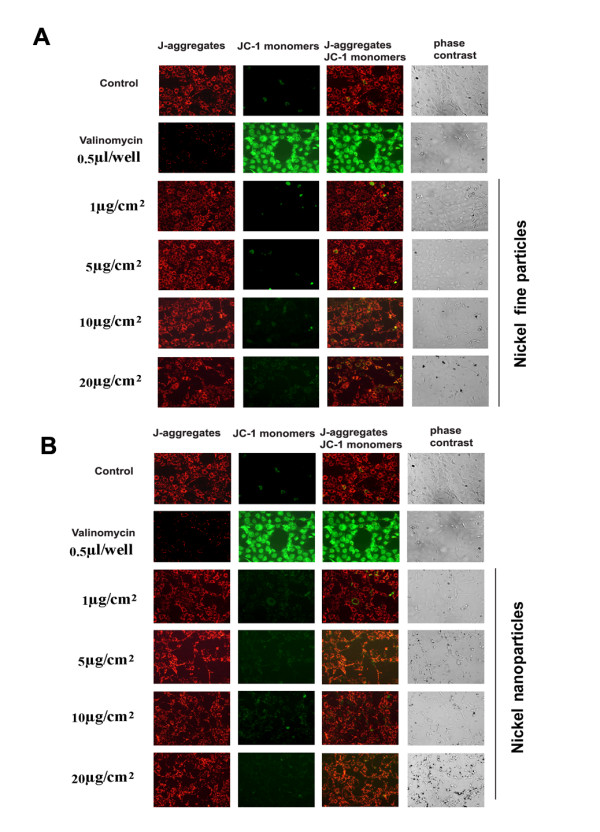
**Effect of metallic nickel particles on mitochondrial membrane permeability**. JB6 cells were treated with various concentrations of metallic nickel nano- or fine particles for 24 h. A mitochondrial staining kit was used to detect the mitochondrial membrane permeability induced by metallic nickel fine (**A**) or nanoparticles (**B**).

Mitochondrial membrane permeability change is a hallmark for apoptosis [31]. JB6 cells were treated with/without various concentrations of metallic nickel particles for 24 h. Mitochondrial membrane permeability was evaluated using a mitochondrial staining kit according to the manufacturer's instructions. The results indicated that neither metallic nickel nano- nor fine particles induced any significant change in the mitochondrial membrane permeability compared to negative control after 24 h treatment. Positive control cells treated with 0.5 μl valinomycin/well for 1 h showed a significant effect on the mitochondrial membrane permeability (Figure 6A and 6B).

## Discussion

Nickel and nickel compounds are widely used in industries. In occupational settings, workers are exposed to a variety of nickel compounds, nickel alloys, as well as metallic nickel. About 10% of all the primary nickel produced is used in metallic form [5]. Human exposure to nickel or its compounds has the potential to produce a variety of pathological effects. The most important adverse health effects due to nickel exposure are skin allergies, lung fibrosis, and lung cancer [7].

With the increase use of nanoparticles in modern industries, inhaled nanoparticles are increasingly being recognized as a potential health threat [32]. It is well known that the toxicity of particles to the lung in both occupational and environmental settings is not only related to exposure but also to the particle size. Accordingly, metallic nickel nanoparticles may be more toxic than the conventional metallic nickel fine particles.

In the present study, results show that both metallic nickel nano- and fine particles induce a dose-related increase in cytotoxicity in JB6 cells after 24 h exposure. In addition, metallic nickel nanoparticles are more toxic than fine particles. Our *in vitro *finding is in agreement with the previous *in vivo *reports that metallic nickel nanoparticles are more toxic on the bronchoalveolar lavage fluid in rats than metallic nickel fine particles [9]. Apoptosis is a programmed form of cell death which is now widely recognized as being of critical importance in health and disease. Although studies have demonstrated that nickel compounds induce cell apoptosis [12], the molecular pathways have not been well investigated. It is generally accepted that cell death can either result in apoptosis or necrosis. Our results suggest that both metallic nickel nano- and fine particles induce JB6 cell death through apoptosis, but not necrosis, at early exposure time in a certain dose range. With the treatment duration prolonged or treatment dose enhanced, both metallic nickel nano- and fine particles can induce JB6 cells necrosis or late apoptosis. For the quantification of apoptosis, we carried out YP staining to determine the apoptotic cells induced by various concentrations of metallic nickel particles. The results showed that both nano- and fine particles induce JB6 cell apoptosis in a dose response manner after 24 h treatments in a dose range of 0.1–20 μg/cm^2^. At concentrations of 5 μg/cm^2^, the number of apoptotic cells induced by nanoparticles was 4 fold higher than fine particles. Our results suggest that both metallic nickel nano- and fine particles are cytotoxic in JB6 cells, while metallic nickel nanoparticles show higher cytotoxicity and apoptosis induction than fine particles. In an inhalation study in rats, Oberdörster *et al *found TiO2 nanoparticles to be more inflammatory than fine particles [11]. When normalized to surface area, the authors found that the dose-response curves for the nano- and fine particles were similar, suggesting that the pulmonary inflammation was mediated by surface effects. In the present study, surface area of metallic nickel nanoparticles is 11-fold greater than fine particles. However, metallic nickel nanoparticles exhibited potency for toxicity and apoptosis which was somewhat less than 11-fold greater than fine particles. Therefore, surface area tends to over correct for the greater toxicity probably because it over estimates the surface area of agglomerates.

In mammals, signaling cascades culminating in apoptotic cell death can be divided into intrinsic or extrinsic pathways. The extrinsic pathway is activated upon ligation of death receptors. The intrinsic pathway can be initiated by cellular stresses such as cytochrome *c *release from mitochondria into the cytoplasm. Our results indicate that metallic nickel particles induced Fas, FADD, DR3, and caspase-8 up-regulation. DISC formation by Fas, FADD and caspase-8 was also found. The formation of the DISC signaling platform may play an important role in the process of activation of caspase-8. Our results suggest that, in the apoptotic process induced by metallic nickel particles, the extrinsic signal pathway is initiated. To investigate the involvement of intrinsic pathways in the apoptotic process induced by metallic nickel particles, cytochrome *c *release was examined. Our results show that, although both metallic nickel nano- and fine particles produced up-regulation of cytochrome *c *in the mitochondria, no obvious cytochrome *c *release was detected in the apoptotic process. In addition, mitochondrial permeability assay show that neither metallic nickel nano- nor fine particles induced significant changes in the mitochondrial membrane permeability, which was in parallel with the Western blot results. These results indicate that the apoptotic process induced by metallic nickel particles is initiated by a cytochrome *c*-independent pathway.

Lamins are nuclear membrane structural components that are important for maintaining normal cell functions. Proteolysis of lamins has been observed in different cells undergoing apoptosis [33]. Degradation of lamina proteins can be triggered by both the extrinsic and the intrinsic pathways [34]. Our results show that lamin A was cleaved in JB6 cells treated with nickel particles, suggesting its involvement in the apoptotic process.

Major cytoskeletal filaments, such as actin, can be degraded during the execution phase of apoptosis [35]. The actin cytoskeleton is capable of responding to complex signaling cascades. Recent studies suggest that it may play key roles in regulating apoptosis [36]. Reports indicate that disruption of actin filament integrity promptly induces apoptosis in adherent epithelial cells [37]. In addition, the dynamic state of actin is important in the regulation of ion channels [36]. In the present study, both metallic nickel nano- and fine particles induce beta-actin cleavages after 1 h treatment in JB6 cells. Our data, combined with a report by Steven *et al *[37], suggest that the actin cytoskeletal network may act as a target of apoptosis and an early signaling component toward apoptotic commitment in the apoptotic process induced by metallic nickel particles.

Recent studies have showed that activation of caspase-8 leads to cleavage of BID. Cleavage of BID translocates from the cytoplasm to the mitochondria and to induce cytochrome *c *release [30,38]. The Bcl-2 proteins are a family of proteins involved in the response to apoptosis. Some of these proteins such as Bcl-2 are antiapoptotic, while others are proapoptotic. In the present study, neither metallic nickel nano- nor fine particles induced any obvious cytochrome *c *release. These results suggest that increased Bcl-2 and down-regulated BAX proteins may antagonize the effects of activated BID on the translocation of cytochrome *c*. In addition, accumulating evidence suggests that Akt activation is sufficient to inhibit the release of cytochrome *c *from mitochondria by up-regulating Bcl-2 protein expression and the alterations in the inner mitochondrial membrane potential [23]. In this study, Akt was activated by metallic nickel particles. Except for the inhibitory effect on the release of cytochrome *c *from mitochondria into cytoplasm, Akt activation may provide the necessary conditions for the selection of cells that have become resistant to apoptosis, which may also be important in the metallic nickel-induced carcinogenic process. Therefore, further research is needed to elucidate the role of activation of Bcl-2 and Akt in the carcinogenicity of metallic nickel particles.

The execution of apoptosis comprises both caspase-dependent and caspase-independent processes. AIF was identified as a major player in caspase-independent cell death. AIF is ideally located in the mitochondria to perform a vital normal function in energy production. Translocation of AIF from mitochondria to the cytoplasm can induce cell apoptosis [39]. Evidence shows that the release of AIF is secondary to both activation of caspase-8 and increasing translocation of BID [39]. In the present study, both metallic nickel nano- and fine particles induced mitochondrial AIF up-regulation and release from mitochondria into the cytoplasm after 1 h treatment. Furthermore, our findings imply that AIF release may occur independently from changes in mitochondrial inner membrane permeability. It has been reported that after an apoptotic insult, changes in the mitochondrial outer membrane permeability may be enough to induce AIF release from mitochondria into the cytoplasm and the nucleus [40]. It may also be possible that AIF is released from mitochondria into the cytoplasm through a specific channel [41]. Our results are in agreement that the fast release of AIF from mitochondria into cytoplasm is preceded by increasing of the proapoptotic Bcl-2 family member BID.

Caspases, a family of aspartic acid-specific proteases, are the major effectors of apoptosis. In the present study, caspase-3, -6 and -9 were only slightly activated in the apoptotic process induced by metallic nickel nano- or fine particles. Interestingly, metallic nickel particles induced caspase-3 precursor up-regulation. Our results suggest that apoptosis induced by metallic nickel nano- or fine particles may be mainly through a caspase-independent pathway.

Mitochondria play an important role in the regulation of cell death. Changes in the mitochondrial membrane permeability are considered an early event in apoptosis. Many proapoptotic proteins can be released from the mitochondria into the cytoplasm, following the formation of a pore in the mitochondrial membrane. In this study, neither metallic nickel nanoparticles nor metallic nickel fine particles induced significant changes in the mitochondrial membrane permeability in JB6 cells after 24 h treatment. These findings imply that, unlike AIF release, cytochrome *c *release is through a mitochondrial membrane permeability change-dependent manner.

In summary, the major findings of the present study are that metallic nickel nanoparticles elicit higher cytotoxicity and apoptosis induction than fine particles. We also identified that metallic nickel particles could induce JB6 cell death through apoptosis, but not necrosis after 24 h treatment in a dose range of 0.1–20 μg/cm^2^. To our knowledge, this is the first study showing that metallic nickel particles activated Fas, FADD, caspase-8, and induced BID cleavage. We provided evidence for DISC formation of Fas-FADD-caspase-8. Another notable finding is that AIF, but not cytochrome *c*, is released from mitochondria into the cytoplasm in the apoptotic process in JB6 cells induced by metallic nickel particles. Notably, upon activation of apoptosis induced by metallic nickel particles in JB6 cells, no significant changes of mitochondrial membrane permeability could be detected. Our results demonstrate that a caspase-8/AIF mediated cytochrome *c*-independent pathway may play a major role in metallic nickel particle-induced apoptosis.

## Abbreviations

MTT: 3-(4,5-dimethylthiazol-2-yl)-2,5-diphenyltetrazolium bromide; FADD: Fas-associated protein with death domain; DR3: death receptor 3; DR6: death receptor 6; IP: Immunoprecipitation; DISC: death-inducing signaling complex; AIF: apoptosis-inducing factor; p-Akt: phospho-Akt; TNF-R2: tumor necrosis factor-receptor 2; IARC: International Agency for Research on Cancer; EMEM: Eagle's minimal essential medium; FBS: fetal bovine serum; DMSO: dimethyl sulfoxide; YP: YO-PRO-1; PI: propidium iodide; SEM: scanning electron microscopy.

## Competing interests

The authors declare that they have no competing interests.

## Disclaimer

The opinions expressed in this article are those of the authors and do not necessarily represent any agency determination or policy.

## Authors' contributions

JZ, LB, XZ and BJ performed the majority of the experiments. JZ, LB, XS, VC and MD involved in writing the manuscript and designing the overall project. All authors read and approved the final manuscript.
